# Generation and characterization of *Aldh3-Cre* transgenic mice as a tool for conditional gene deletion in postnatal cornea

**DOI:** 10.1038/s41598-020-65878-1

**Published:** 2020-06-03

**Authors:** Sweetu Susan Sunny, Jitka Lachova, Naoko Dupacova, Anna Zitova, Zbynek Kozmik

**Affiliations:** 1 Laboratory of Eye Biology, Institute of Molecular Genetics of the Czech Academy of Sciences, Division BIOCEV, Prumyslova 595, 252 50 Vestec, Czech Republic; 2 Laboratory of Transcriptional Regulation, Institute of Molecular Genetics of the Czech Academy of Sciences, Videnska 1083, Praha 4, 142 20 Czech Republic

**Keywords:** Developmental biology, Genetics, Molecular biology

## Abstract

Conditional gene targeting in mice by means of Cre-loxP strategy represents a powerful approach to study mammalian gene function. This approach is however dependent on the availability of suitable strains of mice with a tissue or time restricted activity of the Cre recombinase. Here we describe *Aldh3-Cre* transgenic mice as a useful tool to conditionally delete genes in cornea, a specialized transparent tissue found on the anterior-most part of the eye, which acts as a protective barrier and contributes to the refractive power. Using a set of floxed alleles we demonstrate high Aldh3-Cre activity in corneal epithelial cells, corneal stroma and conjunctival epithelial cells at postnatal stages. Aldh3-Cre will thus be particularly beneficial for functional analysis of genes which are vital for postnatal development of cornea and conjunctiva.

## Introduction

Vision is a complex process which begins with the refraction of light through the cornea, a highly specialised tissue with unprecedented transparency, refractive and protective properties. In mouse, the adult cornea comprises of mainly three layers, anterior non-keratinised stratified squamous epithelium which is 6–8 cell layers thick, collagenous stroma with sparsely dispersed keratocytes, and posterior monolayered endothelium^1,2^. Corneal morphogenesis involves differentiation of surface ectoderm to two-layer thick corneal epithelium and migration of neural crest-derived mesenchymal cells to space between the lens and corneal epithelium, which forms stromal keratocytes and endothelium^3,4^. Corneal epithelium continues to develop after birth; coincident with eye-opening corneal epithelium cells proliferate and differentiate to form 4–5 layered epithelium by postnatal day (PN)21 and form matured 6–8 layered tissue by eight weeks after birth. In the mature cornea, superficial cells of corneal epithelium are regularly sloughed off and constantly replaced by apically moving differentiated basal cells, which in turn are maintained by limbal epithelial stem cells present in limbus which forms a transition between cornea and conjunctiva^5–7^. Any abnormalities in the development and maintenance of cornea result in loss of corneal transparency and thus reduced visual acuity^8^.

Classical gene knockout mouse models are excellent tools for studying gene function *in vivo*, but embryonic lethality or developmental arrest complicates the dissection of gene function during postnatal stages^9,10^. In contrast, the generation of conditional gene knockout mice using the Cre-*loxP* system resolved these difficulties. In conditional gene knockout models, spatiotemporal expression of Cre driver controls Cre mediated inactivation of the gene of interest^11,12^.

Various Cre driver lines are available for conditional gene deletion in the ocular surface. However, these driver lines start Cre-mediated recombination during embryonic development^13–18^. In the case of genes which are essential for embryonic and postnatal corneal development, it makes it difficult to determine whether the defects in maturation and self-renewal of the cornea is due to abnormal development or gene function at the postnatal stages. Hence the generation of Cre driver line, which is restricted to postnatal corneal development would permit a more precise understanding of gene function.

Aldehyde dehydrogenase III (Aldh3), encoded by *Aldh3A1* gene, constitutes nearly one half of total water-soluble protein fraction in the mammalian adult cornea^19^. Aldh3 plays a vital role in protecting the eye from ultraviolet radiation as well in maintaining corneal transparency^20,21^. Endogenous expression of Aldh3 starts at PN9 in very low levels and increases robustly by PN13 in corneal epithelial cells of mouse cornea, coincident with eye-opening^22^. This expression pattern suggests Aldh3 as a promising candidate for driving the Cre expression to cornea specifically at postnatal stages. In the present study we generated BAC transgenic mice expressing Cre recombinase under cis-regulatory control of *Aldh3* gene. We characterised the Cre expression pattern as well as the efficiency of Cre-mediated recombination. Our data suggest that Aldh3-Cre is a very useful tool for postnatal deletion in case of genes which specifically expressed only in the corneal epithelial cells.

## Results

### Generation of *Aldh3 -Cre* transgenic mice and characterisation of Cre activity using *Rosa26R* reporter strain

**Figure 1:**
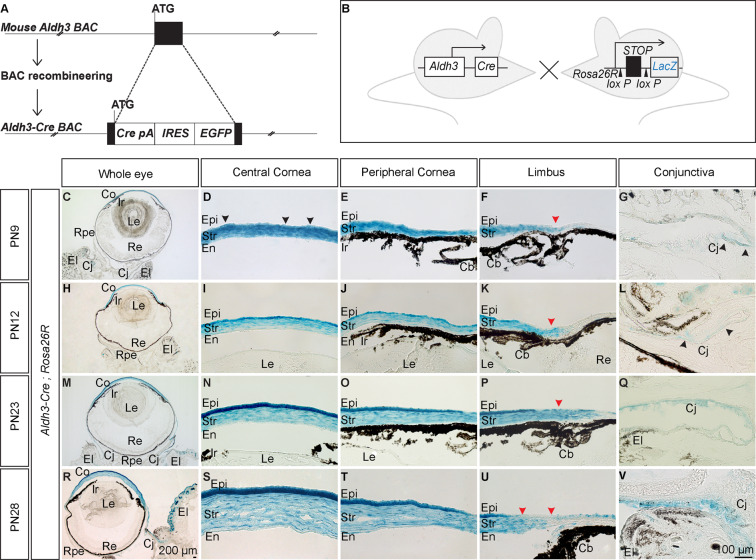
Generation of *Aldh3-Cre* BAC transgenic mice and characterisation of Cre expression pattern. (**A**) Schematic representation of the modification of a BAC clone containing *Aldh3* gene by BAC recombineering. A cassette containing the coding sequence of *Cre* recombinase (Cre-pA) and the coding sequence of *EGFP* linked by *IRES* sequence was inserted into the first translational start site (ATG) of *Aldh3* gene. Black box indicates exons. (**B**) Schematic of mouse lines used to characterise Cre expression pattern. *Aldh3-Cre* driver line was crossed with *Rosa26* reporter strain. This reporter strain has stop cassette flanked by *loxP* sites precedent to *LacZ* gene. In the presence of Cre recombinase, the *LacZ* gene will be expressed upon deletion of *loxP* flanked stop cassette. (**C–V**) Coronal section of the eye at indicated stages was stained with X-gal to show Cre activity in the ocular surface. (**F,K,P,U**) Red arrowheads indicate regions without Cre activity in the limbus region. (**D,G,L**) Black arrowheads indicate few lacZ^+^ ve cells in corneal epithelial cells and conjunctival epithelial cells. Scale bar −100 µm except for (**C,H,M,R**) −200 µm. Abbreviations used in this figure and consecutive images: Co, Cornea; Le, Lens; Re, Retina; Rpe, Retinal pigment epithelium; El, Eyelid; Epi, Corneal epithelium; Str, Corneal stroma; En, Corneal endothelium; Cb, ciliary body; Ir, Iris; Cj, Conjunctival epithelium.

To generate *Aldh3-Cre* transgenic mice, we used a large BAC-based construct harbouring regulatory sequences of *Aldh3A1* gene. A cassette carrying Cre recombinase and EGFP coupled via internal ribosomal entry site (IRES) was introduced into the translation start site of the *Aldh3A1* by BAC recombineering, and the modified BAC clone was then used for pronuclear injections (Fig. 1A). Founders were screened for the presence of the BAC and a single transgenic line was established by breeding to *C57Bl/6*. To visualise the Cre recombinase activity, we mated *Aldh3-Cre* transgenic mice with *Rosa26R* reporter strain. In *Aldh3-Cre; Rosa26R* double transgenic mice, Cre induced recombination activated the expression of *LacZ* under *Rosa26* promoter by excision of *loxP*-flanked stop cassette. (Fig. 1B)^23^. As a result, X-gal staining in *Aldh3-Cre; Rosa26R* mice reveals the spatio-temporal activity of Cre *in vivo*.

Beginning by embryonic day (E)15.5, weak X-gal staining was found in few cells in the presumptive corneal stroma (Supplementary Fig. S1). By PN6 X-gal staining spreads over the anterior corneal stroma (Supplementary Fig. S1). Later on, by PN9 the Cre activity starts in few corneal epithelial cells, in addition to intense X-gal staining throughout the corneal stroma (Fig. 1C–F). A more conspicuous X-gal staining becomes apparent in corneal epithelial cells by PN12 (Fig. 1H–J) with a further sharp increase from PN12 to PN28 (Compare Fig. 1H–J with M-O, R-T). Besides this, we observed mosaic X-gal staining in conjunctival epithelial cells in PN9 and PN12 (Fig. 1G,L) and considerably stronger expression in later postnatal stages (Fig. 1Q,V). In accordance with the previous report^24^, we found weak or no X-gal staining in limbal epithelial cells in all indicated stages (Fig. 1F,K,P,U). We found no X-gal staining on age-matched *Rosa26R* mice, used as negative controls, upon parallel incubations (Supplementary Fig. S1).

Our experiments demonstrated the spatio-temporal expression pattern of Aldh3-Cre, in which highly mosaic Cre activity was found at embryonic stages, whereas a strong rate of recombination was detected at postnatal stages in the cornea, concurrent with eye-opening.

### Ectopic expression of β-catenin using Aldh3-Cre results in the formation of corneal epithelial nodules

**Figure 2:**
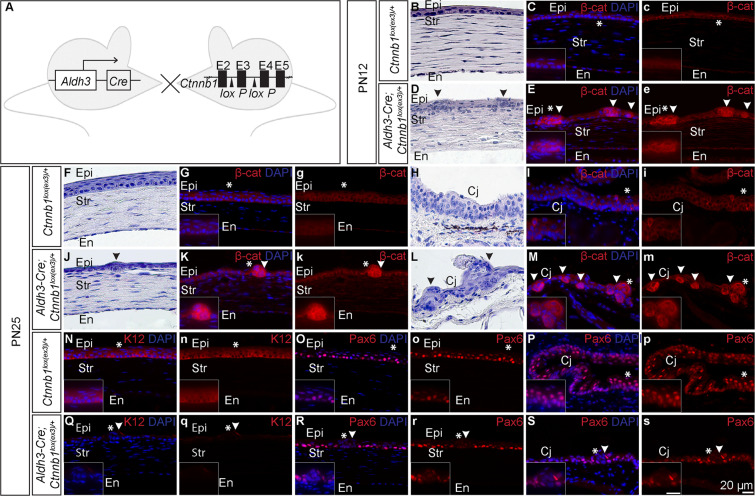
Ectopic expression of β-catenin using Aldh3-Cre results in epithelial hyperplasia. (**A**) Schematic of mouse lines used for ectopic expression of *Ctnnb1* gene. Generation of *Aldh3-Cre; Ctnnb1*^*lox(ex3)/*+^ double transgenic mice, by crossing *Aldh3-Cre* driver line with *Ctnnb1*^*lox(ex3)/*+^ transgenic mice, which harbours *loxP* sites flanking exon3 of *Ctnnb1* gene. Cre mediated deletion results in the accumulation of a stabilised Ctnnb1 protein in the cells. (**B–S**) Corneal sections from PN12 and PN25 of wild type and gain of function mutant were subjected to H & E staining and antibodies indicated. Epithelial protrusions (arrowheads) were found on (**B–K**) corneal and (**L,M**) conjunctival epithelial cells of gain of function mutant. (**N,Q**) Loss of K12 expression in the entire corneal epithelium of gain of function mutant. Diminished Pax6 expression in epithelial protrusions found on (**O,R**) corneal and (**P,S**) conjunctival epithelium. Inserts are the higher magnification of corresponding panels and asterisks (*) indicates its position. Scale Bar- 20 µm.

In order to evaluate the utility of Aldh3-Cre in conditional gene modification, we crossed *Aldh3-Cre* transgenic mice with a mouse strain that harbours a conditional allele of *β-catenin*, *Ctnnb1*^*lox(ex3)/*+^ (Fig. 2A). Upon Cre-mediated recombination *loxP* sites flanking exon3 of *Ctnnb1* gene will be excised which results in mutant β-catenin protein resistant to phosphorylation and proteasome-mediated degradation. This stabilised mutant β-catenin protein accumulates in the cell and mimics activation of canonical Wnt signaling (β-catenin gain-of-function situation)^25^. For the sake of simplicity, now onwards we refer *Ctnnb1*^*lox(ex3)/*+^ as wildtype and *Aldh3-Cre; Ctnnb1*^*lox(ex3)/*+^ as a gain-of-function mutant in the subsequent text. To investigate the effect of ectopic β*-catenin* expression in the ocular surface of the gain-of-function mutant we performed morphological analysis of different developmental stages from PN6 to PN25. We observed no conspicuous changes in the ocular surface until PN9 (Supplementary Fig. S2). However, epithelial protrusions began to appear in the corneal epithelium of gain of function mutant from PN12 (Fig. 2D,J) and conjunctival epithelium by PN25 (Fig. 2L), whereas age-matched wildtype exhibited normal corneal (Fig. 2B,F) and conjunctival epithelium (Fig. 1H). This observation is consistent with a previous study showing that the gain-of-function of β-catenin induces hyperplastic transformation in corneal epithelial cells^26^.

In addition to the phenotype described above, H & E staining also revealed that unlike wildtype, which possessed 5–6 layers at PN25 (Fig. 2F), the gain-of-function mutant exhibited thinner epithelium with only 2–3 layers (Fig. 2J). This phenotype corroborates previous study showing that the aberrant β-catenin expression in stromal keratocytes prevents corneal stratification^27^.

To confirm that phenotypic consequences in the cornea of β-catenin gain-of-function mutant are due to the increased level of β-catenin we performed immunohistochemistry staining using an antibody that recognises C-terminus of the protein. Indeed, we confirmed that compared to wildtype, there was a significant increase of β-catenin staining in corneal epithelial cells and stroma from PN12 (Fig. 2C,E,G,K) and conjunctival epithelial cells by PN25 (Fig. 2I,M).

Next we investigated whether corneal epithelial differentiation is affected upon β-catenin overexpression. Immunohistochemistry staining showed diminished expression of Pax6 in the epithelial nodules formed in the corneal (Fig. 2O,R) and conjunctival epithelium (Fig. 2P,S) of the gain-of-function mutant. Furthermore, in contrast to wildtype corneal epithelium which exhibited a typical keratin 12 (K12) expression (Fig. 2N), the gain-of-function mutant completely lost K12 expression in the entire corneal epithelium (Fig. 2Q). This data indicates that corneal character is lost upon β-catenin activation, as reported previously^26^.

Combined, our data suggest that Aldh3-Cre mediated recombination is high in the corneal stroma, corneal epithelial cells and conjunctival epithelial cells postnatally. Furthermore, Aldh3-Cre is capable of efficiently generating stabilised β-catenin protein upon breeding to *Ctnnb1*^*lox(ex3)*^ to replicate previously described phenotypes.

### Postnatal inactivation of β-catenin in cornea using Aldh3-Cre

**Figure 3:**
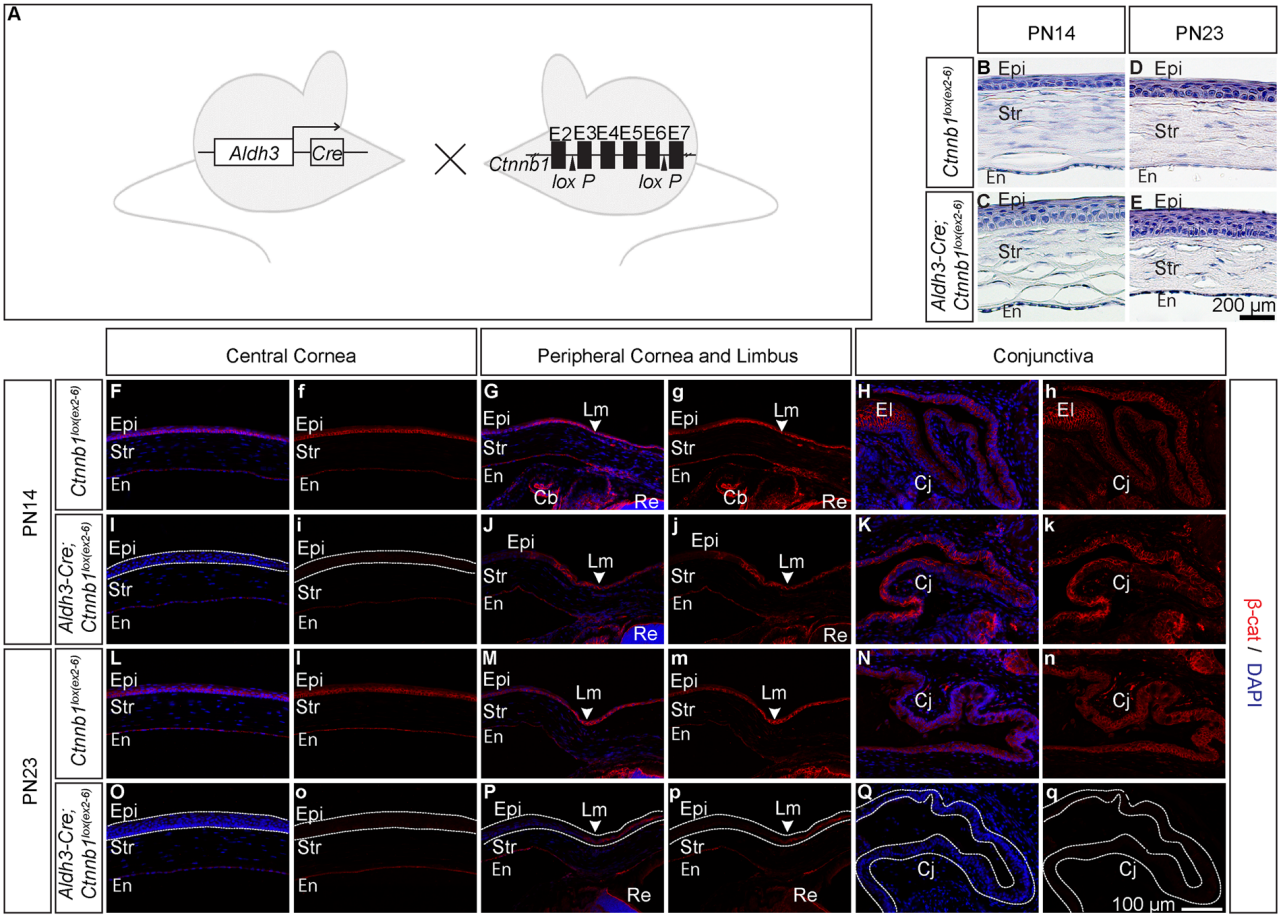
Postnatal deletion of β-catenin using Aldh3-Cre. (**A**) Schematic of mouse lines used for conditional inactivation of β-catenin. (**B–E**) H & E staining on corneal sections of wildtype and loss of function mutant from PN14 and PN23 old mice. Corneal epithelium of loss of function mutant had more layers at PN14 and PN23 in comparison to age-matched control epithelium. (**F–Q**) β-catenin staining on corneal and conjunctival sections from PN14 and PN23 old mice of wild type and loss of function mutant. (**F,I,L,O**) Loss of β-catenin in corneal epithelial cells from PN14 and (**H,K,N,Q**) conjunctival epithelial cells by PN23.(**G,J,M,P**) Depletion of β-catenin in the peripheral cornea at indicated stages, but note that staining retained in limbal epithelial cells. The dashed line indicates a region with β-catenin deletion. White arrowheads indicate the position of the limbus. Abbreviations: Lm, Limbus. Scale bar (**B–E**) −200 µm (**F–Q**) −100 µm.

To further assess the efficacy of Aldh3-Cre in conditional gene deletion, we generated *Aldh3-Cre; Ctnnb1*^*lox(ex2–6)/lox(ex2–6)*^ mice, in which *loxP* sites flank exons 2–6 of the *β-catenin* gene (Fig. 3A)^28^. Cre mediated deletion removes exons 2–6 of the floxed allele and results in depletion of β-catenin in the cells. For the sake of simplicity, from now onwards we refer to *Aldh3-Cre; Ctnnb1*^*lox(ex2–6)/lox(ex2–6)*^ as a loss-of-function mutant and *Ctnnb1*^*lox(ex2–6)/lox(ex2–6)*^ as a wildtype. Immunohistochemistry staining showed depletion of β-catenin in corneal epithelial cells of loss-of-function mutant compared to wildtype by PN14, whereas we found no significant decrease in β-catenin staining in limbal epithelial cells and conjunctival cells at this stage (Fig. 3F–K). However, we observed a notable depletion of β-catenin protein in corneal and conjunctival epithelial cells by PN23 (Fig. 3L–Q). As expected, even at PN23 we found no evidence of β-catenin gene deletion in limbal epithelial cells (Fig. 3M,P). Next we determined the phenotypic consequence of β-catenin gene deletion in *Aldh3-Cre; Ctnnb1*^*lox(ex2–6)/lox(ex2–6)*^ mice. Histological analysis of postnatal corneas at PN14 and PN23 showed that unlike control littermates the loss-of-function mutants have thicker epithelium (Fig. 3B–E). This result is in agreement with the previous findings that the loss of β-catenin in stromal keratocytes results in precocious stratification^29^. Taken together, these experiments demonstrated that Aldh3-Cre-mediated excision of β-catenin floxed allele resulted in the depletion of β-catenin protein from postnatal corneal and conjunctival epithelial cells. Combined, our data suggest that Aldh3-Cre is a useful tool for gene modification for elucidating biological functions of any gene of interest during postnatal corneal (or conjunctival) development.

## Discussion

Based on serial analysis of gene expression data (SAGE) demonstrating that *Aldh3* is one of the most abundant genes in the postnatal cornea^30^, we generated an *Aldh3-Cre* transgenic driver mouse line for conditional gene modification in the postnatal cornea. We characterised the expression pattern of Cre recombinase by X-gal staining after breeding *Cre* driver line with *Rosa26R* reporter mice. Endogenous expression of Aldh3 protein starts at PN9^22^ and is restricted to corneal epithelial cells of the mouse cornea^24^. In contrast, we observed mosaic Cre activity in the corneal stroma by E15.5 (Supplementary Fig. S1) and strong Cre activity throughout the corneal stroma by PN9 (Fig. 1C–V). We also noted that X-gal staining starts in few corneal epithelial cells at PN9, and becomes stronger coincidentally with eye-opening, as described previously^22^. BAC transgenes carry extensive cis-regulatory sequences of the corresponding gene, and thus usually Cre recombinase activity recapitulates endogenous gene expression. One possible explanation for the difference that we noted between the endogenous Aldh3 expression and Cre activity is that the BAC clone used by us does not possess all of the regulatory information of the *Aldh3A1* gene.

We further assessed the functionality of Aldh3-Cre by generating mouse strains *Aldh3-Cre; Ctnnb1*^*lox(ex3)/*+^ and *Aldh3-Cre; Ctnnb1*^*lox(ex2–6)/lox(ex2–6)*^. In both cases, we found that Aldh3-Cre-mediated recombination resulted in excision of the floxed genomic region, and generated a gain- and loss-of-function mutant for β-catenin, respectively, that recapitulated previously described phenotypes (Figs. 2 and 3). A recent study showed a constitutive expression of active β-catenin in postnatal corneal epithelial cells and dynamic expression in the corneal stroma during PN1 to PN21^29^. This expression pattern will make it difficult to determine whether the phenotypic consequences on corneal epithelial cells are precisely due to loss/gain of β-catenin in corneal epithelial cells or due to its loss/gain in the corneal stroma. Despite this limitation, *Aldh3-Cre* strain described here is a highly efficient driver line for postnatal gene ablation in case of genes which are specifically expressed in corneal epithelial cells. Furthermore, Aldh3-Cre is a useful tool for deletion of genes that cause developmental arrest during embryonic development, allowing to determine their function in the postnatal stages. Finally, we propose that the late onset of Cre expression in conjunctival epithelial cells will be beneficial for the functional analysis of genes which are vital for its postnatal development.

## Methods

### Ethics statement

Experimental mice were housed and *in vivo* experiments were executed in compliance with the European Communities Council Directive of 24 November 1986 (86/609/EEC) and national and institutional guidelines. Experimental procedures for handling the mice were approved by the Animal Care Committee of the Institute of Molecular Genetics (no. 71/2014). This work did not include human subjects.

### Mouse strains

A 164 kb bacterial Artificial Chromosome (BAC) (RP24–338F21) harboring the mouse *Aldh3A1* gene was purchased from Children’s Hospital Oakland Research Institute. To generate *Aldh3-Cre* BAC, the open reading frame of Cre recombinase fused to EGFP via IRES was inserted into the exon 1 containing the translation initiation codon of *Aldh3A1* using a method of BAC recombineering^31^; http://web.ncifcrf.gov/research/brb/protocol.aspx). The CreIRESEGFP-FRT-neo-FRT targeting cassette was PCR-amplified from pCS- CreIRESEGFP-FRT-neo-FRT using *Aldh3* forward and reverse targeting primers: 5′-gccatcctccttcctctgatgcaaagtgttctctatccccagttaccatgtccaatttactgaccgtaca-3′; 5′-gcgctgcaacgcctccagctgctcaacccggaactgcagcggtcgagtctgctattccagaagtagtgag-3′. The PCR product was purified using QIAEX Gel extraction Kit (Qiagen) and treated with DpnI to destroy the template plasmid. The PCR product was electroporated into bacterial strain EL250 carrying RP24–338F21 BAC, and colonies were tested for homologous recombination by PCR. The kanamycin resistance cassette was next removed by induction of flipase activity in EL250 cells. Modified *Aldh3-Cre* BAC DNA was isolated and used for pronuclear injection. Mice were genotyped using primers that recognize the recombination junction, with forward primer located upstream of the Aldh3 translation start site (5′-aattagcatagtggtggagtca-3′) and reverse primer located in Cre recombinase coding region (5′-cgttgcatcgaccggtaatgca-3′). For analysis of Cre activity, the animals of the tenth and later generations from the original founder were used for genetic crossing. Samples exhibited reproducible Cre-mediated recombination regardless of the breeding generation.

Following are the other genetically modified mouse strains used for this study; *Rosa26R*^23^ (Jackson Laboratory, stock no. 003309), *Ctnnb*^*lox(ex3)*^^25^, and *Ctnnb*^*lox(ex2–6)*^^28^. The genotype of each mouse was identified by PCR analysis of tail DNA.

### X-gal staining for LacZ activity

We harvested mouse eyes at different developmental stages from PN9. Enucleated eyes were fixed in 0.2% formaldehyde in 1x PBS for 15 min at room temperature on ice, washed 2 × 5 minutes with the rinse buffer (0.1 M phosphate buffer pH 7.3, 2 mM MgCl_2_, 0.01% sodium deoxycholate and 0.02% Nonidet P-40), followed by incubation in X-gal staining solution (rinse buffer,5 mM potassium ferrocyanide, 5 mM potassium ferricyanide, 20 mM Tris pH 7.3, 1 mg/ml X-gal) for 1 hour at 37 °C and overnight at room temperature. Eyes were postfixed in 4% formaldehyde in 1x PBS overnight at 4 °C, cryoprotected in 30% sucrose and frozen in OCT (Tissue Tek, Sakura). Frozen tissues were sectioned at 18 µm thickness.

### Histology and Immunohistochemistry for corneal characterisation

Enucleated eyes were fixed in 4% formaldehyde in 1x PBS for overnight at 4 °C, washed 3 × 10 minutes in 1x PBS and followed by paraffin embedding. For histology analysis, paraffin sections (6 µm) were deparaffinised, rehydrated with ethanol series and stained with Hematoxylin and Eosin (H & E). For immunofluorescence, paraffin sections (6 µm) were deparaffinised, rehydrated with ethanol series, followed by antigen retrieval in sodium citrate buffer (10 mM sodium citrate buffer, 0.05% Tween 20, pH-6.0). The sections were blocked in 10% BSA/0.1% PBT for 30 minutes and incubated with primary antibodies in 1% BSA/0.1% PBT for overnight. On the following day, samples were washed in 1x PBS for 3 × 10 minutes, incubated with secondary antibodies in 1% BSA/0.1% PBT for 1 hour, washed in 1x PBS, counterstained with DAPI (1 µg/ml) and mounted in Mowiol (Sigma). Following are the antibodies used for this study: rabbit anti-β-catenin (1:1000, Sigma, C2206), rabbit anti- Pax6 (1:1000, Covance, PRP-278P), goat anti-K12 (1: 300, Santa Cruz Biotec Inc, Sc-17101), anti-rabbit Alexa 594, and anti-goat Alexa 594 (all 1:500, Molecular Probes). Stained sections were analysed with Zeis Imager Z2 (Zeis, Germany).

## Supplementary information

Supplementary information.

## Data Availability

All the data generated and analysed in our study are included in this article.
