# Supplementary material for: Generation and characterization of Aldh3-Cre transgenic mice as a tool for conditional gene deletion in postnatal cornea
Source: Sci Rep. 2020 Jun 3;10:9083. doi: 10.1038/s41598-020-65878-1 (PMC7270111; doi:10.1038/s41598-020-65878-1)
Supplement: Supplementary file 1 — Supplementary information. [file 41598_2020_65878_MOESM1_ESM.pdf]

Supplementary Information

Generation and characterization of *Aldh3-Cre* transgenic mice as a tool for conditional gene deletion in postnatal cornea.

Sweetu Susan Sunny[1], Jitka Lachova[1], Naoko Dupacova[1], Anna Zitova[1,2] and Zbynek Kozmik[1,2*]

[1] Laboratory of Eye Biology, Institute of Molecular Genetics of the Czech Academy of Sciences, Division BIOCEV, Prumyslova 595, 252 50 Vestec, Czech Republic

[2] Laboratory of Transcriptional Regulation, Institute of Molecular Genetics of the Czech Academy of Sciences, Videnska 1083, Praha 4, 142 20, Czech Republic

[*] Corresponding author: Institute of Molecular Genetics of the Czech Academy of Sciences, Videnska 1083, Praha 4, 142 20, Czech Republic

E-mail: kozmik@img.cas.cz

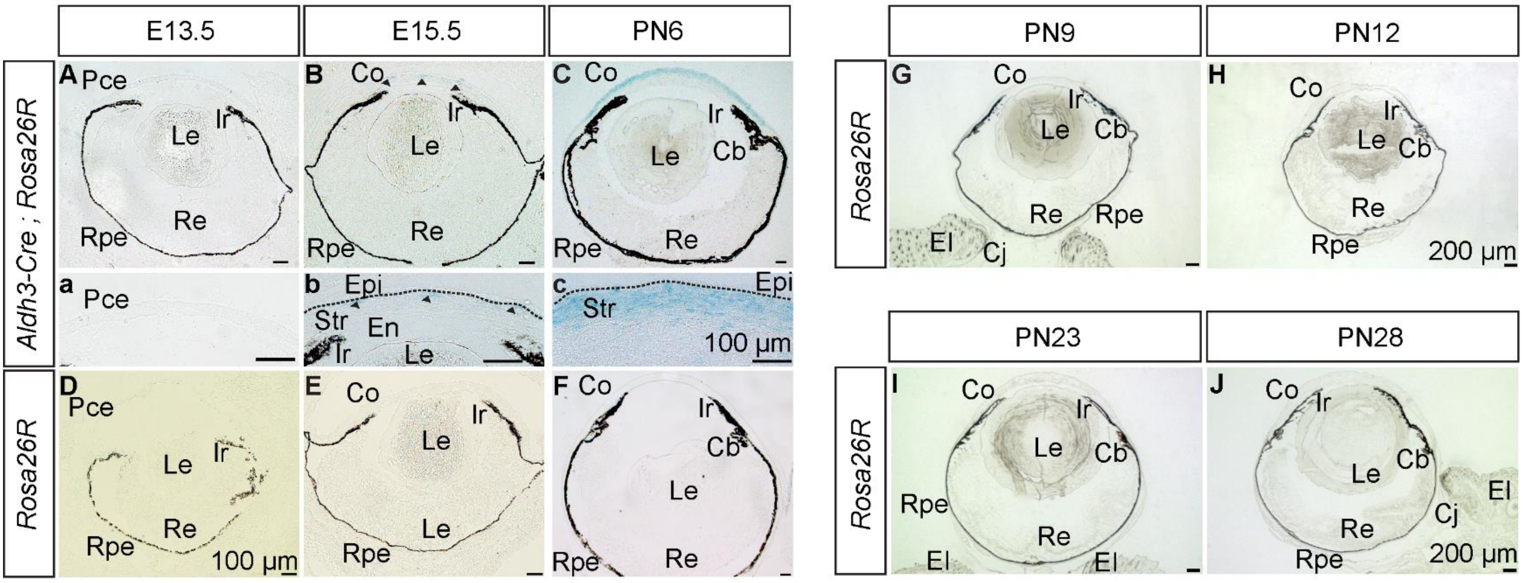

Supplementary Figure S1. **Mosaic Cre activity starts at E15.5 and no endogenous lacZ expression upon X-gal incubation**. (A-C) X-gal staining on frontal sections from indicated stages revealed (B, b) mosaic Cre activity on corneal stroma (black arrowheads) at E15.5 and (C, c) more spreaded activity by PN6 (D-J) No Cre activity in age-matched *Rosa26R* sections and (A,a) sections from E13.5. Abbreviations: Pce, Presumptive corneal epithelium. Scale bar: (A-F) - 100 µm, (G-J) - 200 µm

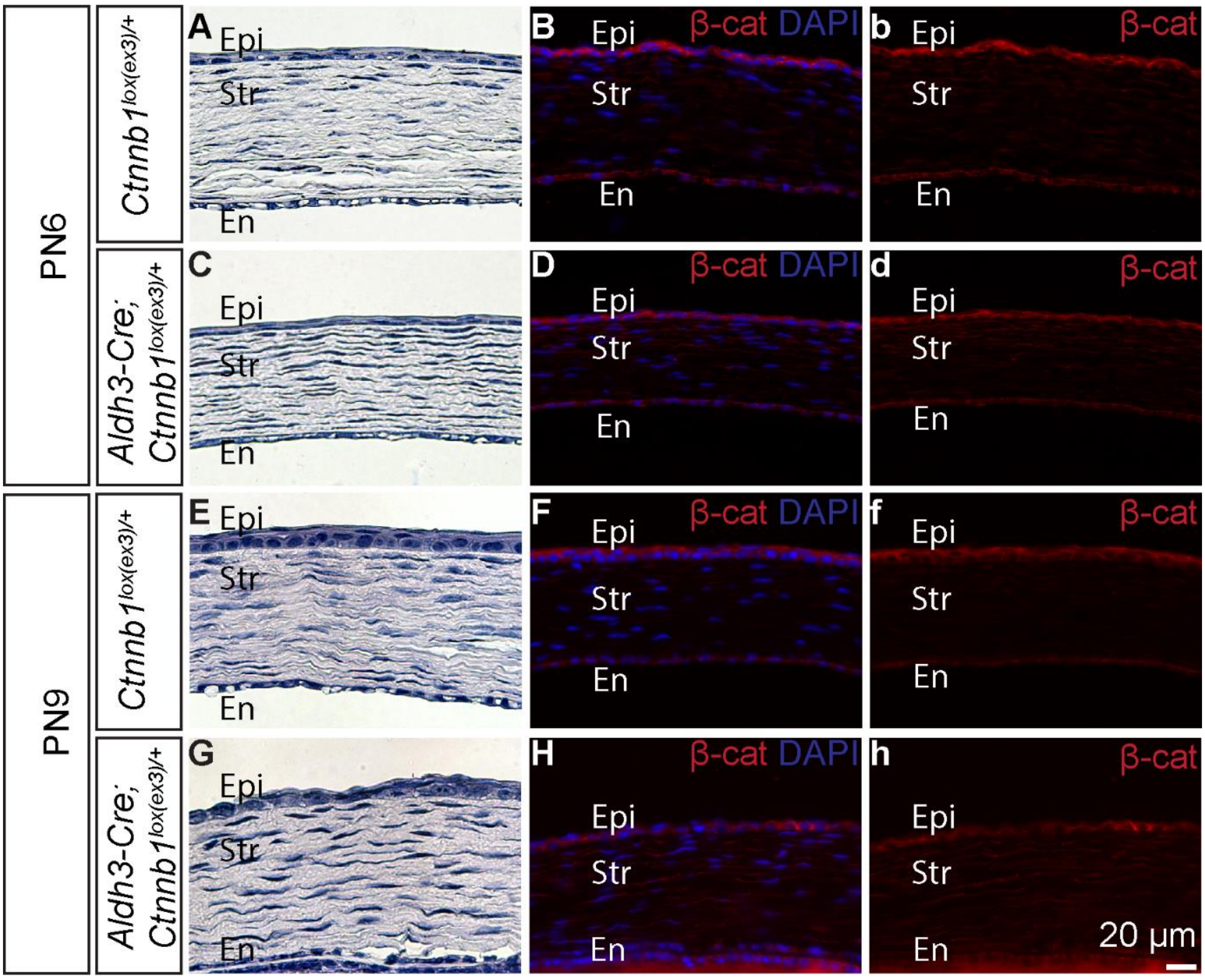

Supplementary Figure S2. **No epithelial protrusions until PN9 upon ectopic β-catenin activation**. (A-H) H & E staining and immunostaining with β-catenin on corneal sections from PN6 and PN9. (A, C, E, G) No significant morphological changes till PN9. (B, D, F, H) No significant increase in β-catenin levels in corneal epithelial cells, but there is an increase in the corneal stroma at PN9. Scale bar- 20μm
